# Neural Cell Interactions with a Surgical Grade Biomaterial Using a Simulated Injury in Brain Organotypic Slices

**DOI:** 10.3390/jfb15120362

**Published:** 2024-11-30

**Authors:** Jessica Patricia Wiseman, Divya Maitreyi Chari

**Affiliations:** 1 School of Medicine, Keele University, Keele ST5 5BG, UK; 2 Faculty of Biology, Medicine and Health, University of Manchester, Manchester M13 9PL, UK

**Keywords:** biomaterials, scaffold, traumatic brain injury, organotypic slices, 3D model

## Abstract

Tissue engineering research for neurological applications has demonstrated that biomaterial-based structural bridges present a promising approach for promoting regeneration. This is particularly relevant for penetrating traumatic brain injuries, where the clinical prognosis is typically poor, with no available regeneration-enhancing therapies. Specifically, repurposing clinically approved biomaterials offers many advantages (reduced approval time and achieving commercial scaleup for clinical applications), highlighting the need for detailed screening of potential neuromaterials. A major challenge in experimental testing is the limited availability of neuromimetic, technically accessible, cost-effective, and humane models of neurological injury for efficient biomaterial testing in injury-simulated environments. Three dimensional (3D) organotypic brain slices bridge the gap between live animal models and simplified co-cultures and are a versatile tool for studies on neural development, neurodegenerative disease and in drug testing. Despite this, their utility for investigation of neural cell responses to biomaterial implantation is poorly investigated. We demonstrate that murine brain organotypic slices can be used to develop a model of penetrating traumatic brain injury, wherein a surgical-grade biomaterial scaffold can be implanted into the lesion cavity. Critically, the model allowed for examination of key cellular responses involved in CNS injury pathology/biomaterial handling: astrogliosis, microglial activation and axonal sprouting. The approach offers a technically simple and versatile methodology to study biomaterial interventions as a regenerative therapy for neurological injuries.

## 1. Introduction

Biomaterial-based scaffolds have emerged as a significant platform for promoting neural repair by acting as structural bridges, offering numerous advantages that are highly desirable in implanted devices. These include ease of delivery to injury sites, the capacity to offer physical support to host tissue including cell infiltration, and to match the biochemical/biophysical environment of the brain along with the ability to deliver therapeutic agents [1]. Biomaterials such as hydrogels and electrospun nanofibre scaffolds have been tested for regeneration in brain injuries with encouraging outcomes such as modulation of the injury environment (altered astrocyte and inflammatory responses), and structural support to nerve fibres [1]. For translational applications, there are major benefits in repurposing pre-approved neurosurgical biomaterials [2] for different neurological applications to reduce costs and regulatory burden, as well as the time and complexity in bringing a new therapeutic product to market. 

Accordingly, the use of biomaterials for neural regeneration is the focus of intensive research in neural tissue engineering. However, a barrier to translation in the development and testing of novel biomaterials (e.g., hydrogels, nanoparticle platforms, nanofibre constructs) is the need for cost-effective, technically facile, humane and injury mimetic tissue models (representative of three-dimensional (3D) brain tissue architecture) for biomaterial screening. Live animal models of neurological injury are among the most invasive procedures in experimental science, often causing significant neurological deficits and raising ethical concerns, in addition to requiring specialised approvals and incurring high costs. By contrast, simpler reductionist models like two-dimensional (2D) cell cultures, while easier to maintain and analyse, lack the representative neural architecture and functional complexity of in vivo tissue.

In this context, organotypic slice cultures (OSCs) of the brain can provide a versatile bridge between isolated cell cultures and in vivo experiments [3]. While offering a ‘reductionist’ approach that allows for ease of manipulation and analysis, these models still retain the complex cytoarchitecture and structural relationships of neural tissue in vivo. They have been used widely for long term, high throughput neuroscience and drug testing assays. Various ages, brain anatomical areas and species, including human foetuses and transgenic models can be used as tissue donor sources, offering high flexibility for experimental studies depending on the specific application. Additionally, these models are amenable to electrophysiology and molecular biology analyses, as well as a range of cell imaging techniques [3]. Various models of traumatic injury have been described in organotypic slices including tissue stretch/strain, compression, blast models using compressed air and weight drop [4,5,6,7]. 

Despite these advantages, the use of organotypic slices for biomaterial testing is a relatively under-studied area. Weightman et al. showed the feasibility of implanting a poly-lactic acid nanofibre scaffold over transecting injuries in mouse organotypic spinal cord slices, with dramatic alignment of neurons, astrocytes and microglia on the scaffolds [8]. The same paradigm could be used to study uptake of magnetic particles in spinal cord lesions, with the microglia (immune cells) dominating particle uptake versus other neural cell types [9]. In 2023, Tickle et al. used transecting injuries in adult human brain slices (derived from surgically resected cerebellar tonsillar tissue from patients with Chiari malformation); key pathological events such as cell scarring, and immune cell infiltration could be replicated in vitro. Furthermore, the study showed that lesional implantation of the surgical grade, Food and Drug Administration (FDA) approved matrix Duragen™ was feasible. However, logistical considerations and the limited availability of surgically resected tissue, precluded a more detailed analysis of neural cell-biomaterial interactions in this study [10]. 

The benefits of using organotypic models for biomaterial screening from such studies are clear. Additionally, when considering the neural processing of biomaterials in model systems, it is crucial that the latter offer the capacity to evaluate bidirectional interactions between glial cells (in particular, astrocytes and microglia) with introduced biomaterials. Arguably, biomaterial handling and processing by these cells is one of the most critical determinants of biomaterial fate in the brain. For example, fibrillar contraction by astrocytes can alter the biomechanical properties of implanted materials, but these cells also show potentially pro-regenerative phenotypes in response to biomaterials [11,12]. The microglial cells resident in the brain have vital roles in the surveillance of the neural microenvironment; they can engulf and degrade biomaterials, impacting their lifespan, functional utility and toxicity responses. However, inflammatory responses can also be modulated by biomaterials [13], altering neuroinflammatory processes. 

To address these issues, this study aims to evaluate the utility of a brain organotypic slice injury model in studying neural cell responses to an implanted clinical grade biomaterial. The objectives are to (i) establish the feasibility of introducing a 3D Duragen™ (DG) implant into a transecting lesion in a brain organotypic slice culture (bOSC); (ii) analyse the responses of microglia and astrocytes at the slice–material interface to evaluate the impact on the astroglial and neural immune response; and (iii) assess the material’s neuroregenerative capacity for transected neurons. Our data highlight the advantages and versatility of the slice injury approach to studying neuromaterial handling in neural injury sites. 

## 2. Materials and Methods

### 2.1. Generating Brain Organotypic Slices, Introducing Injury and Biomaterial Implantation

#### 2.1.1. Brain Dissection from P1–4 Mice

Fresh tissues were dissected from mouse pups (CD1), with litters ranging from eight to 12 pups. Keele University retains Home Office licensed authority as a designated premises, providing regulatory compliance for the care and welfare of the animals used in this study (Keele University Establishment licence number: X350251A8 (copy available on request)). Ethical approval for the Schedule 1 usage of the animals used in this study was obtained from the Keele University Animal Welfare and Ethical Review Body in 2017. Mice maintaining specified pathogen free health status were housed and bred in the Keele Biological Service Unit, in accordance with the Code of Practice for the Housing and Care of Animals Bred, Supplied or Used for Scientific Purposes. Litters were maintained on a continuous 12:12 light cycle, 22.5 ± 0.4 °C, and 46% ± 5% humidity. Mice were bred and maintained according to the UK Code of Practice for the housing and care of animals used for scientific procedures, Animals (Scientific Procedures) Act 1986. Pups of both sexes were used in the study and culled via the schedule 1 method of an overdose of anaesthetic, sodium pentobarbital (Animalcare Ltd., York, UK), 1 mL/kg intraperitoneal injection, on postnatal day 1–4, weight ca 2.5–3.5 g. Brains were dissected and transferred to a dissection medium (2.5% HEPES in Earle’s balanced salt solution) on ice. 

Brain organotypic slice culture and lesioning methods were adapted from Weightman et al., 2014 [8]. For brain dissection, the skull was revealed via an incision through the skin from neck to nose using dissection scissors. Next, a central incision through the skull bone from the back of the skull to the nose was made using micro-dissecting Vannas spring scissors. Lateral incisions were made from the central incision to create flaps in the skull. Using the spatula, the skull bone flaps were lifted, and the brain was removed carefully ensuring no damage to the brain tissue. The brain tissue was immediately placed in the slicing medium (Earle’s balanced salt solution (EBSS) [Gibco, #14155063] buffered with 2.5% of 1 M HEPES [Gibco, #11560496]) on ice. 

#### 2.1.2. Generating Brain Tissue Slices

The brain was cut down the sagittal midline with a scalpel and sliced using a McIlwain Tissue Chopper (Model TC752, Campden Instruments, Loughborough, UK) along the coronal plane (350 µm thickness). The brain slices were immediately placed in a fresh slicing medium (50% EBSS and 50% minimum essential medium (MEM) (Gibco, Thermo Fisher Scientific, Loughborough, UK, #11095080)) and kept on ice for 90 min. At 90 min, the slices were transferred to pre-cut Omnipore ‘confetti’ membranes (JHWP04700), resting on Millicell culture insert membranes (PICM0RG50) inside small round petri dishes (one slice per confetti, three confetti per insert) with culture medium (50% MEM, 25% heat-inactivated horse serum, 25% EBSS and supplemented with 36 mM D-glucose, 0.5% pen/strep). A wide-bore plastic Pasteur pipette (trimmed to widen the bore) was used to facilitate the transfer of a slice to the membrane, and any excess slicing medium was removed through a narrow-bore Pasteur pipette and then a P10 pipette. The pre-cut confetti for each slice allows for the maneuverability of individual slices. This set up generates an air–medium interface between the culture medium and the humidified atmosphere; the medium provides adequate nutrition to the tissue slice through capillary action, whilst the air regulates appropriate gaseous exchange throughout the slice. Slices were cultured for up to 18 days in vitro. Five small petri dishes were placed inside a large square petri dish for ease of transportation and improved sterility; these were incubated at 37 °C, 5% CO_2_. Following 4 days in vitro, a transecting lesion was introduced to mimic a penetrating neurological injury. Every two days, a 50% medium change was carried out.

#### 2.1.3. Introducing a Transecting Lesion in Slices

A double-bladed scalpel tool was assembled with a distance between each blade of 300 µm (as described in [8]), meaning a reproducible lesion size of approximately 300 µm would be produced when introducing the transection injury to the bOSCs. Lesions were carried out under a dissection microscope in sterile conditions. The waste tissue from the injury was removed and any debris was cleared via the aspirator with a fine nozzle to leave a clean transecting lesion, to allow for efficient analysis of the cellular response in the slices. 

#### 2.1.4. Biomaterial Implantation into the Lesion Site

DuraGen™ (DG) was implanted within an hour of lesioning. DG is a porous substrate that absorbs the medium and swells in size; therefore, before application of the material to the lesion site, the material was soaked in the medium. The biomaterial is supplied sterile, and to prepare DG for implantation, a small section (~5 mm × 5 mm) was cut from the provided sheet with a scalpel. This was then transferred to the pre-set tissue chopper under sterile conditions with a small drop of medium, and then sliced at a thickness of 200 µm (thickness optimised for imaging purposes). One sheet was then transferred back to the tissue chopper and chopped to a width of 300 µm (approximate lesion distance). In preparation for lesion width variations, the material was also cut at varying diameters (smaller or larger than 300 µm). The sliced material was then transferred to a small petri dish of media, sealed with parafilm, and kept at 4 °C until further use. Post-injury, the biomaterial was implanted within one hour of injury, and this procedure was carried out under a dissection microscope in sterile conditions. As the transection length can vary between brain slices, DuraGen™ was cut to size with a scalpel to match the length of the injury area before implantation. Both injury only and injury + DG slices were maintained in the same conditions throughout.

### 2.2. Live/Dead and Immunofluorescence Staining

#### 2.2.1. Slice Viability Assessment

Prior to fixation, live/dead assays were carried out at 11 DIV, 18 DIV, 4 DIV + 7 days post lesion (DPL) and 4 DIV + 14 DPL, which involved submerging the slices in a solution of 3 µL/mL ethidium homodimer (Sigma-Aldrich, Burlington, MA, USA, #E1903) to label dead cells and 1 µL/mL of Calcein AM (VWR, Lutterworth, UK, #89139-470) to label live cells in a culture medium for 40 min at 37 °C. The slices were then mounted on a glass slide with Vectashield mounting medium with DAPI (4′,6-diamidino-2-phenylindole) [Vector laboratories, Newark, CA, #H-1200-10] for imaging. Fluorescence micrographs of live/dead staining were captured with consistent exposure settings. 

#### 2.2.2. Immunofluorescence Staining

At appropriate time points post lesioning (4, 7 or 14 days for microglial analysis and 14 days for neuronal and astrocytic analysis), slices were fixed with 4% paraformaldehyde (PFA) in PBS for 1 hr. After fixation, slices were washed with phosphate buffered saline (PBS) three times and incubated with blocking buffer (5% normal donkey serum and 0.3% Triton-X-100 in PBS) at room temperature (RT) for 1 h. Following this, samples were incubated with the appropriate primary antibodies diluted in blocking buffer overnight at 4 °C: mouse anti-Tuj-1 (β-tubulin 3) (BioLegend, San Diego, CA, USA #801202) (1:500), rabbit anti-GFAP (glial fibrillary acidic protein) (BioLegend #644702) (1:500) and goat anti-Iba1 (ionised calcium-binding adaptor molecule 1) (Abcam, Cambridge, UK #ab5076) (1:200). Slices were washed three times with PBS, 5 min per wash, and incubated with the appropriate secondary antibodies diluted in blocking buffer (1:200) for 4 h at RT (or overnight at 4 °C) in dark conditions: FITC donkey anti-goat IgG, FITC donkey anti-mouse IgG and CY3 donkey anti-rabbit IgG (Jackson Immunoresearch Laboratories, West Grove, PA, USA, #705-095-003, #715-095-150, #711-165-152). Subsequently, slices were washed three times with PBS (5 min/wash) and mounted on glass slides with Vectashield^®^ mounting medium (containing DAPI for the nuclear stain). Mounts were sealed with varnish and allowed to dry before imaging. 

All images of brain organotypic slices were taken under a fluorescence microscope (Leica DMC 2500 LED equipped with a CCD camera, DFC350 FX from Leica, Wetzlar, Germany). The software used for imaging was Leica Application Suite X v.1 (2017). Z-stack images for microglia morphological analysis were taken on a confocal microscope (Zeiss, Axioscope A1, Carl Zeiss MicroImaging GmbH, Goettingen, Germany).

### 2.3. Glial Scar Width and Astrocyte Reactivity Analysis

The width of the glial scar was determined using the measuring tool in Fiji (ImageJ), with at least 10 measurements taken per lesion per slice. For each biological replicate, three technical replicates were performed, and the average glial scar thickness from each technical replicate was calculated. 

The level of GFAP expression in activated astrocytes was measured using optical density (OD) calculations. An astrocytic edge from which the OD measurements began was defined as the glial scar-like lesion edge at 0 µm into the slice for the injury-only group, and a varying astrocytic edge for the injury plus DG implantation group. Ingrowth of astrocytes into the material meant that the lesion edge varied from the original incision margin (depicted in Figure S1).

Images were captured with a Leica DMC2500 LED microscope at constant exposure throughout the experiments for reproducible data analysis. For GFAP quantification all images were converted to greyscale (8-bit) and inverted on image J. Quantification of GFAP was achieved by evaluating the optical density profile from the lesion margins towards the centre of the slice, up to 500 µm, each at 100 µm intervals. This was done by drawing multiple parallel lines from the astrocytic edge (Figure S1B,C). ImageJ micrographs were converted to 8-bit type and inverted, and the programme was calibrated to calculate global optical density. Following this, a grid was laid over the image to give the measurement intervals. Parallel lines were drawn from where the grid lines first meet the astrocytic edge and 500 µm into the slice, and these were angled in two directions (Figure S1B,C). Measurements of GFAP OD at 400–500 µm within the slice were assumed to be the baseline astrocytes. A background OD was calculated by measuring the OD of an area with no tissue, which was subtracted from the OD values from the same image to give a true OD. ODs were averaged and a fold change was calculated at each 100 µm interval from the astrocytic edge. 

### 2.4. Microglial Infiltration and Morphology Analysis

Quantification of the microglial cell number within the injury site ± DG was achieved by counting the number of Iba1 positive cells within a given area. Five randomly located fields of view within the lesion area, with or without biomaterial implantation, were taken at 40× magnification per biological repeat. An average value was generated and statistically analysed at 4, 7 and 14 days post lesioning (*n* = 3).

Microglial morphology was characterised by a cell roundness index (CRI) (adapted from [14]). This can quantitatively describe the effect of the biomaterial on the morphology of the resident microglia, to determine their possible reactive state. Iba1 immunolabeled micrographs were imported into ImageJ. The relevant scale was set globally, and the freehand drawing tool function selected. Each Iba1+ cell within the lesion area was traced around and measured; a minimum of 20 cells were measured per biological repeat. Recorded measurements from each traced cell provide the cell perimeter and area values. These values were then plugged into the equation for CRI. CRI = (4 × 3.142 × area)/perimeter^2^), where the value of 1 denotes a complete circle, and towards 0 is ramified (Figure S2). An average microglial CRI was calculated per condition per biological repeat (*n* = 3). 

### 2.5. Statistical Analysis

In all cases the number of experiments, *n*, refers to experiments obtained from separate mice litters. GraphPad Prism v5.0 software was used for all statistical analyses. All values were expressed as the mean ± the standard error of the mean (SEM). In the case of the GFAP analysis, where optical density was measured at increasing distances from the lesion edge, both injury-only and injury + DG were statistically evaluated using a two-way ANOVA. Both microglial cell infiltration and morphology were also analysed using a two-way ANOVA with Tukey’s multiple comparisons test. Tests for normality were carried out per analysis: the F test for variance comparison showed the results from the Iba-1 data set had significantly different standard deviations, meaning a Welch’s T-test (an equal variance *t*-test) had to be carried out, which assumed the standard deviations from each population were not equal. However, there was no significant difference in the standard deviations of the other data sets.

## 3. Results

### 3.1. bOSCs Show High Cell Viability up to 18 DIV, in Control and Lesioned Slices

**Figure 1:**
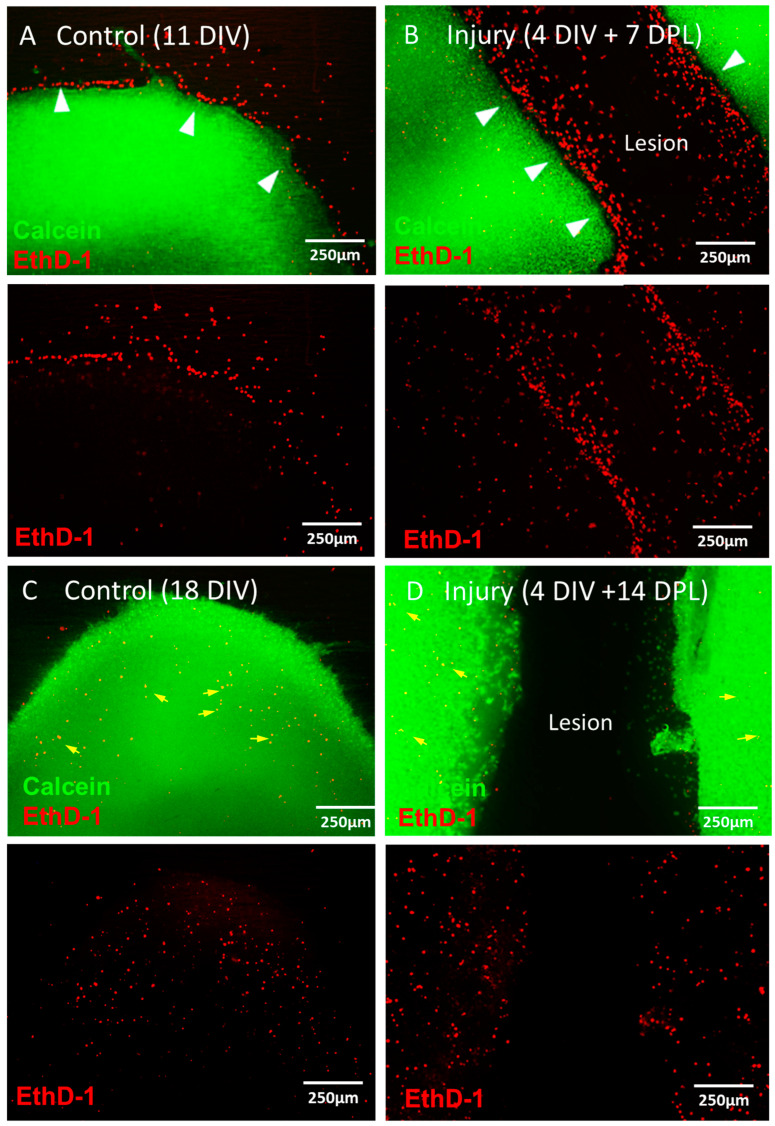
Viability of control and injured bOSCs. Representative micrographs of control, uninjured bOSCs at 11 DIV (**A**) 4 DIV + 7 DPL (**B**), 18 DIV (**C**) or 4 DIV + 14 PDL (**D**). Merged images are shown are for calcein (live cells) and EthD-1 (dead cells), with the corresponding EthD-1 only stain below the corresponding merged image. White arrows highlight dead cells localised to the slice edge of the lesion margins; yellow arrows indicate cell death within the body of the slice at later timepoints.

A complete transecting injury was reproducibly generated within the brain slices. To assess slice viability over time, staining was performed for live (calcein) and dead (ethidium homodimer-1 (EthD-1)) cells at 11 days in vitro (DIV), 18 DIV, 7 days post lesioning (DPL) and 14 DPL (where slices are lesioned at 4 DIV). Initially, cell death post-slice sectioning was observed mainly at the slice periphery, while the main body of the slice showed high viability (Figure 1A). Although the live/dead proportions could not be quantified due to the dense packing of cells, visual inspection of the main body of control slices suggested ~95% slice viability. Similarly, initial cell death post-lesioning was localised to the lesion boundary (Figure 1B), with an estimated viability of 90–95% in the main body of the slice. By 18 DIV/14 DPL, cell death at the slice periphery/lesion margins appeared to diminish. A small number of dead cells could be observed in the slice body at this time point compared to 11 DIV/7 DPL slices (Figure 1C,D), however overall viability was still estimated to be ~85–90%. 

### 3.2. A Hallmark Glial Scar-like Feature Observed in Injured bOSCs at 14 DPL, with Morphological Disruption of the Scar Following Biomaterial Implantation

**Figure 2:**
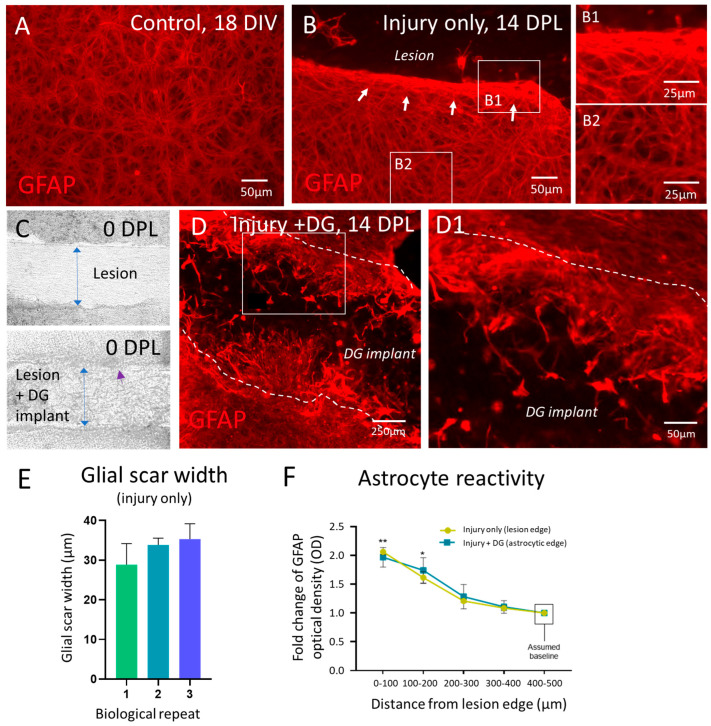
Astrocytes display enhanced GFAP reactivity at the injury site and biomaterial implantation at the lesion supports astrocytic ingrowth (14 DPL). (**A**) Representative micrograph of normal appearing morphologies in a dense syncytium of astrocytes distributed throughout the bOSCs at 18 DIV. (**B**) Representative micrograph of injury-induced astrogliosis at the lesion margin, including a band of intense GFAP expression and a glial-scar like interconnected network of astrocytes at 14 DPL (shown in higher magnification in (**B1**)). (**B2**) displays little or no astrogliosis in astrocytes distant from the lesion margins. White arrows indicate the band of increased GFAP staining. (**C**) Phase contrast images of the lesion area pre- and post-DG implantation (at 0 DPL) where the material can be observed filling the lesion cavity. Blue arrows identify the implanted biomaterial sheet. The purple arrow indicates the biomaterial-slice interface. (**D**) At 14 DPL, there was extensive astrocytic ingrowth into the DG implant, with an apparent disruption of the glial scar-like structure. Astrocytes in the material varied in levels of GFAP expression. The dashed line indicates the lesion margins. (**D1**) Magnified view of ((**D**), white box) displays that astrocytes at the biomaterial–slice interface show varying levels of GFAP expression and a range of cellular morphologies. (**E**) Bar graph presents the average glial scar width per biological repeat. There are no significant differences between data. Data are presented as mean ± SEM and analysed by a one-way ANOVA with Tukey’s multiple comparisons test, (*n* = 3). (**F**) Graph demonstrates a significant increase in GFAP expression (optical density measurements) at the lesion margins of injury-only slices compared to the baseline GFAP expression 400–500 µm from the lesion site. Injury + DG implantation showed no obvious enhancement or reduction in GFAP reactivity. Data are presented as mean ± SEM and analysed by a two-way ANOVA with Tukey’s multiple comparisons test, *n* = 4, asterisks indicate * *p* < 0.1, ** *p* < 0.01.

Glial scar formation in response to injury and therapeutic biomaterial implantation must be assessed to establish any pro-regenerative or pro-scarring potential of the introduced material. Uninjured control slices showed a dense astrocyte network at 18 DIV with stellate morphologies (Figure 2A). Injured slices showed evidence of astrogliosis and formation of a glial scar-like structure at the lesion margins at 14 DPL, associated with a dense band of increased GFAP expression and cellular hypertrophy (Figure 2B), which was also observed at the slice periphery (data not shown). Within 1 h of injury, the neurosurgical grade biomaterial DG could be carefully introduced into the lesions of the treatment group (Figure 2C). Following implantation, there was a clear disruption of the band of astrocytic scarring and cells showed extensive ingrowth into the biomaterial (Figure 2D). Some astrocytes within the material showed high levels of GFAP expression, suggesting a reactive phenotype. Higher magnification of the lesion edge of injured bOSCs + DG clearly shows astrocytic ingrowth and varying degrees of astrocyte reactivity (Figure 2(D1)). The width of the glial scar was consistent across both technical and biological replicates, with no significant differences between data sets (Figure 2E) (one-way ANOVA, with Tukey’s multiple comparisons, *n* = 3). Optical density measurements of GFAP expression confirmed that injured slices display significantly enhanced GFAP expression levels towards the lesion margins (0–100 µm), with a 2-fold increase (2.06 ± 0.5 OD) compared to the corresponding baseline astrocyte GFAP expression (400–500 µm away from the lesion margin). Biomaterial implantation into the injury site of bOSCs showed a similar 2-fold increase (1.97 ± 0.12 OD) from the baseline level (two-way ANOVA, Tukey’s multiple comparisons, *p* < 0.01 **, *n* = 4, Figure 2F). No difference was observed between OD measurements at the injury-only lesion edge versus the material slice interface. 

### 3.3. Limited Axonal Outgrowth Observed in Injured Slices with and Without Biomaterial Implantation

**Figure 3:**
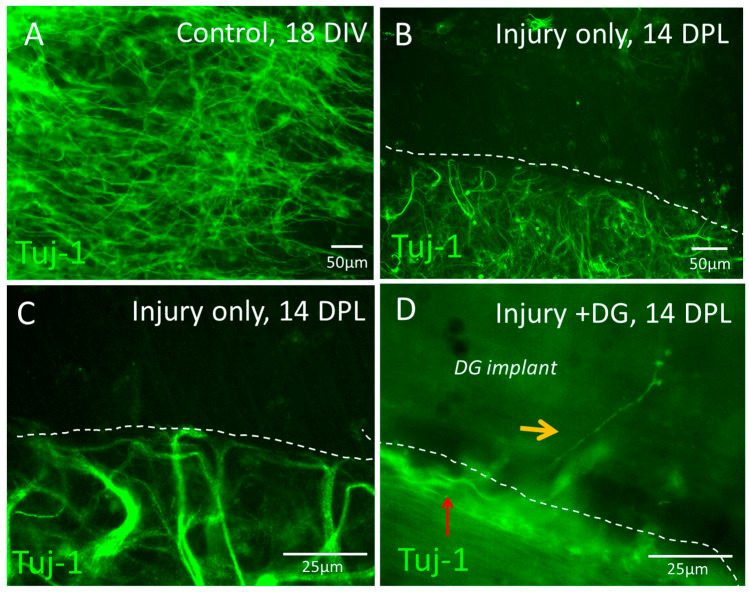
Limited axonal outgrowth observed with or without biomaterial implantation (14 DPL). (**A**) Representative micrograph of the neuronal network within control, uninjured slices (18 DIV). (**B**) Restricted axonal outgrowth was observed from the lesion margins in injury-only slices (14 DPL). (**C**) Higher magnification of (**B**) at the lesion edge confirms lack of axonal outgrowth. (**D**) DG implantation shows limited effects on axonal outgrowth into the material (14 DPL). Orange arrow indicates a single neurite extension into DG. Red arrow indicates majority of neurite growth along the lesion edge within the slice. Dashed lines indicate lesion margins (**B**,**C**) or material boundary (**D**).

The ability of the implanted DG matrix to support axonal growth for functional recovery was assessed in the lesions. At 18 DIV, injured slices displayed an extensive Tuj1+ neuronal network throughout the slice (Figure 3A). Following a transecting injury, injured bOSCs showed no evidence of nerve fibre outgrowth from the lesion margin by 14 DPL, indicating very limited regenerative capacity in the slices (Figure 3B), which was confirmed at a higher magnification (Figure 3C). A small number of nerve fibres were observed to grow into the implanted scaffold; however, this observation was limited and inconsistent (Figure 3D), precluding quantification. Neurite growth within the slices was often observed to align along the lesion edge, rather than extending into the biomaterial. 

### 3.4. Microglia Extensively Infiltrate the Implanted Biomaterial and Exhibit Ramified Phenotypes

**Figure 4:**
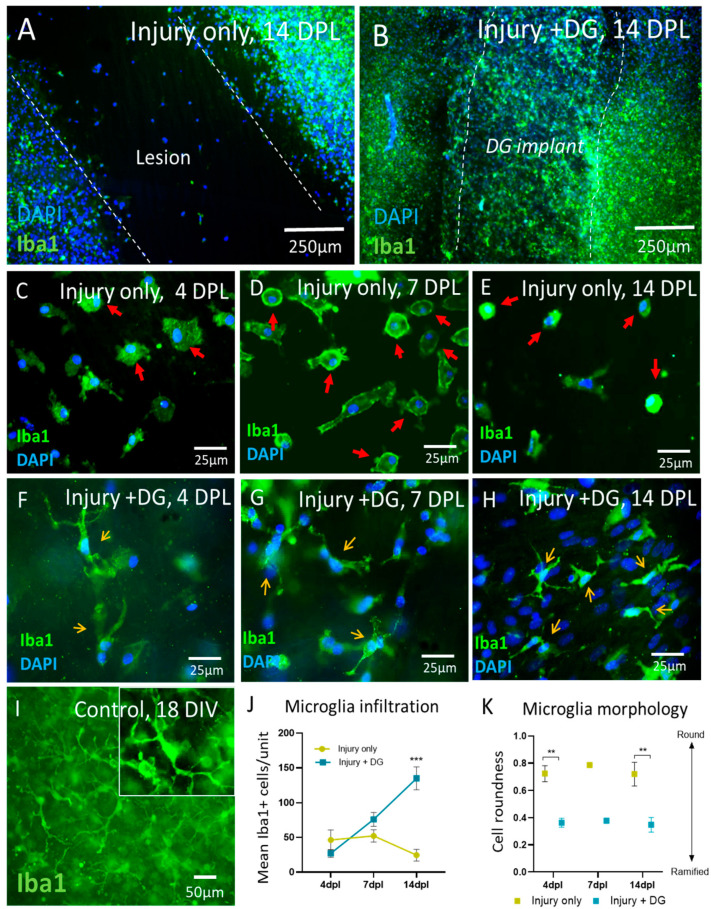
Microglia are observed to robustly colonise the biomaterial. (**A**) Representative micrograph of the injury-only slice, showing a low level of microglial infiltration (14 DPL). Dashed line indicates lesion edge. (**B**) Representative micrograph of the slice injury site with implanted DG, showing extensive microglial infiltration (14 DPL) compared to the injury alone. Dashed line indicates material boundary. (**C**–**E**) show a range of microglial morphologies with amoeboid (rounded) or bushy activated microglial morphologies (red arrows) observed within the injury-only lesion site at 4, 7 and 14 DPL, respectively. (**F**–**H**) demonstrate microglia with a less reactive microglial phenotype and branched morphology within the DG implant at 4, 7 and 14 DPL, respectively. Orange arrows indicate branched ramified-like microglia. (**I**) Ramified ‘resting’ microglial morphology in control uninjured bOSCs at 18 DIV. Inset shows high magnification of ramified phenotypes. (**J**) Graph shows that microglial numbers reduce after 7 DPL in injury-alone slices without biomaterial implantation. With biomaterial implantation, a dramatic increase in microglial infiltration is observed. (**K**) Morphological analysis of microglial phenotypes shows that cells within the DG implant are significantly more ramified than microglial cells within the lesion alone (data presented as mean ± SEM and analysed by a two-way ANOVA with Tukey’s multiple comparisons, ** *p* < 0.01, *** *p* < 0.001, *n* = 3).

Assessment of the immune cell response is an important step in establishing an implanted material’s immunogenic profile. Iba1+ (a microglial cytoskeleton marker) stained a widespread population of microglia within the slices. At 14 DPL, Iba1 staining revealed a low number of microglia infiltrating the lesion site (Figure 4A). In contrast, a significantly higher number of microglia was observed infiltrating the lesion area when the DG implant was present (Figure 4B). An assessment of the morphologies of the material-residing microglia at 4, 7, and 14 DPL showed that they appear to resume a more ramified resting or transitional morphological phenotype compared to the reactive amoeboid or bushy microglia [15] in the injury-only lesion sites (Figure 4C–H). This ramified resting microglial morphology is observed throughout the control slices without injury (Figure 4I) and in injured bOSCs away from lesion sites (data not shown). Quantification of microglial infiltration across the three timepoints 4, 7, and 14 DPL showed an initial influx of microglia at 4 DPL in injury-only slices, which persisted until 7 DPL before beginning to decline in number (46.4 ± 14.2 and 52.2 ± 8.8, at 4 and 7 DPL, then dropping to 24.5 ± 8.4 at 14 DPL). In contrast, bOSC injuries with DG implantation exhibited a similar initial influx at 4 DPL; however, by the 7 and 14 DPL timepoints, microglial infiltration had continued to increase (Figure 4J, two-way ANOVA with Tukey’s multiple comparisons, *n* = 3, microglial numbers rising to 27.0 ± 5.6 at 4 DPL, 76.1 ± 10.0 at 7 DPL and 135.0 ± 16.3 at 14 DPL). Notably, while microglial infiltration was high, the microglia residing within the biomaterial had significantly greater numbers of ramified morphologies than cells within the lesion site alone at corresponding timepoints (microglial CRI in the injury-only group was 0.723 ± 0.034, 0.783 ± 0.011 and 0.720 ± 0.050 at 4, 7 and 14 DPL, respectively, while the CRI of microglia in the injury + DG were 0.361 ± 0.013, 0.376 ± 0.008, and 0.348 ± 0.031 at 4, 7 and 134 DPL respectively) (Figure 4K, two-way ANOVA with Tukey’s multiple comparisons, *n* = 3).

## 4. Discussion

This study demonstrates the potential of a 3D brain slice injury model for investigating neural cell interactions at lesion sites with an implanted, neurosurgical-grade biomaterial. We show that the model is both neuromimetic (with major neural cell populations detected) and pathomimetic (with key neuropathological responses observed). Precise implantation of a biomaterial within the transecting injury cavity enabled reliable assessment of the scaffold’s effects on pathological processes, such as astroglial scarring and immune cell infiltration.

We selected DuraGen™ for implantation as it is an FDA-approved clinical grade biomaterial composed of semi-synthetic, 95% ultra-pure type 1 collagen, widely used in neurosurgery as a dural substitute. DuraGen™ has been designed to limit “immunological response, and foreign body reaction” and is naturally resorbed in 6–8 weeks (https://products.integralife.com/file/general/1518550095.pdf, accessed on 14 October 2024). Previous research has demonstrated that this material is non-toxic and promotes the survival of cortical neurons, wherein glutamate-induced toxicity of cultured neurons on the DuraGen™ matrix showed no adverse effects versus a glass substrate [16]. Finch et al. demonstrated that DuraGen™ can act as an effective encapsulating scaffold, and mouse neural stem cells (NSCs) seeded within the biomaterial matrix had > ca 94% cell viability and could differentiate into appropriate cellular phenotypes within the material. Notably, the material has been implanted into the brain with positive outcomes. Rats subject to cortical impact brain injury with implantation of DuraGen™ over the injury showed a significant enhancement of spatial memory gain (18 days post implantation), but motor function was not affected. The collagen matrix was shown to reduce the contusion lesion area and improve neuronal survival, suggesting that DuraGen™ has a neuroprotective and possibly pro-regenerative influence [17]. These results justify the relevance of this material in the current study, for translational and biomaterial repurposing applications. DuraGen™ was implanted acellular and without any modifications, to study its regenerative permissiveness as a biomaterial alone.

Our data show that pathological mechanisms following biomaterial implantation can be efficiently assessed within our model. Complete transection facilitates analysis of post-injury responses due to ease of lesion localisation, with no residual tissue within the lesion site. For example, axonal regeneration is easier to verify anatomically versus non-penetrating, contusion force or stretch models (due to the incomplete nature of these injuries). Further, creation of an identifiable injury cavity is ideal for implantation of a test biomaterial and allows for straightforward analysis/imaging of cell-biomaterial interactions within the lesion. Approximately 30 viable intact cortical brain slices can be routinely obtained from a P2–P4 mouse litter which permits the simultaneous assessment of several treatment conditions, and various tissue/cellular assessments from a single litter of pups. This mitigates some experimental variability and reduces the number of animals required for therapeutic assessment, thereby addressing the reduction element of the 3R principles of animal usage (reduction, replacement, refinement). 

The mouse bOSCs showed high viability at the time points examined (>85%) and exhibited typical neural cellular phenotypes up to 18 days in vitro (the latest time point examined here). Post-lesion, the slices also displayed hallmark injury responses such as infiltration of activated microglia into the injury cavity, astrogliosis at the injury margins and limited axonal sprouting, mimicking in vivo pathological responses. 

We report a modulation of the glial scar morphology with material implantation, where astrocytes extensively infiltrate the biomaterial. Our study reports neither a reduction nor exacerbation of the glial scar on interaction with the biomaterial, suggesting that the material is safe for implantation. The study of biomaterial implantation in brain organotypic slices is generally limited; however, a 2D in vitro model of pTBI, using a scratch wound assay on cortical glial cultures, showed no significant difference in GFAP expression in peri-lesional astrocytes with or without DG implantation, similar to this study [18]. Most biomaterial implantation studies in central nervous system (CNS) injuries are performed in animal models, typically rodents, and these biomaterials are often functionalised with regenerative molecules or therapeutic cell populations. Many studies indicate a significant reduction in glial scarring following biomaterial implantation [19,20,21,22,23]. A laminin-inspired self-assembling peptide hydrogel, and an anti-inflammatory macromolecule, fucoidan injected into the lesion cavity after brain injury was capable of reducing glial scarring to half that of the injury without treatment, along with a significant change in astrocytic morphology at the lesion site [20]. The use of a collagen-impregnated acrylic hydrogel made of 2-hydroxyethyl methacrylate, implanted into adult rats with spinal cord injury (SCI), resulted in minimal astrocytic reactivity at the material–tissue interface, with no cystic cavitation and axonal penetration along the full length of the hydrogel [23]. In contrast, a collagen scaffold functionalised with hippocampal progenitor cells implanted into a rat model of penetrating brain injury did alter injury-induced glial scarring [24]. In addition to different functionalisation, another key difference in these studies is the timing of treatment relative to injury, with the latter study conducting implantation at 7 days post-initial injury, while the other studies performed it immediately after implantation. This suggests that the implantation timepoint may be a critical factor in attenuating glial scarring, which warrants investigation when screening biomaterials. Further, non-functionalised collagen scaffolds alone generally do not appear to attenuate astrocyte reactivity and may require linkage with functional molecules or cellular components to effectively modulate scarring reactions. 

Our data also demonstrates extensive microglial infiltration into the biomaterial within lesion sites up to 14 DPL, compared to the lesion cavity alone. This observation suggests the material induces an immunogenic response, i.e. recruiting additional microglia to manage the ‘foreign body’, similar to findings in [18]. However, detailed morphological assessments indicate that microglia within the material assume branched, ramified morphologies, which are more typical of ‘resting’ microglia, as opposed to rounded, activated phenotypes. Similar findings were reported in two models of central system injury. One involved an injectable hydrogel in an organotypic spinal cord injury (SCI) model, where microglial activation was reduced, indicated by a decreased microglial perimeter within the injury site; treatment with this hydrogel functionalised with the Epac2 agonist S-220 also further reduced the microglial perimeter [22]. The second study demonstrates that implantation of a gelatin hydrogel into a transecting SCI mouse model increases expression of a ramified microglial phenotype compared to the amoeboid phenotype injury-only control. The numbers of ramified microglia were further enhanced in the lesion site after depleting the activated microglial population, followed by reintroduction of inactivated microglia before implantation of the hydrogel [25]. These findings suggest that ‘soft’ mouldable biomaterials can modulate immune responses in neurological injury sites. When comparing the implantation of soft versus stiff biomaterials in the brain, it is evident that stiffer materials elicit a more pronounced ‘foreign body’ immune reaction [1]. We can speculate that within an injury environment, the soft 3D nature of the implant mimics the stiffness of the endogenous environment of uninjured neural tissue, inducing a change in microglial phenotypic profiles away from a reactive injury-induced state. Assessing microglial morphology provides valuable insights into their reactive state. Microglia can exhibit a range of morphologies, reflecting varying levels of activation, from resting to intermediate activation states to highly reactive. These include ramified (resting state), primed, hypertrophied, bushy, and amoeboid (highly reactive state) [15]. A detailed comparative proteomic study of microglia extracted from soft biomaterial substrates, versus those derived from normal brain tissue, would provide further insights in this regard, along with the immunomodulatory profile of implanted scaffolds. 

Our model also enabled evaluation of the growth of CNS axons into implanted biomaterials. While limited axonal sprouting was occasionally observed in lesions with biomaterial implantation, there was no significant enhancement of outgrowth following the implantation. This is likely due to a lack of axonal growth factors within the biomaterial or inadequate topographical guidance cues for effective neuronal guidance. It is likely that further functionalisation of DuraGen™ with extracellular matrix (ECM) molecules, neurotrophic growth factors (i.e., BDNF) [26,27] or a cellular component (i.e., neural progenitor cells (NPCs)) [28] is needed, in order to enhance axonal regeneration. Our biomaterial is porous with randomly aligned fibres; for effective brain regeneration, smaller electrospun nanofibres with topographical and ECM cues may be more suitable biomaterials for axonal guidance [29,30]. In this case, it appears that the neurosurgical-grade biomaterial would still require further development into a bioactive implant. Nevertheless, the limited axonal regeneration observed highlights the value of our model in specifically screening biomaterials that aim to enhance neurite outgrowth in brain injury sites, including those containing topographical cues to guide outgrowing fibres. 

Our study presents a methodology for the developmental testing and screening of biomaterials, in a moderate throughput approach. Future work will explore a range of neurosurgical-grade materials, both alone and with functional modifications, to improve therapeutic potential. Additionally, incorporating aligned fibres within the biomaterial may better support neuronal reconnection across lesions. In terms of the limitations of the model, it does not currently include circulating immune cells or an interface with a vascular supply/perfusion system, although it should be noted that brain slices have been reported to possess a robust vascular network [31]. It also does not encompass a functional blood–brain barrier (BBB) component. It is feasible to add the former into lesion areas, and innovations from tissue engineering and the use of artificial blood vessels could enhance the functionality of the approach [32]. Moreover, adding a functional assay—where the bOSC can be interfaced with a microelectrode array (MEA) system including conformable electrodes [33]—could offer valuable insights by measuring functional electrophysiological recovery. Interfacing the model with microfluidic systems and model systems to mimic the BBB could further advance the model system [34,35]. 

## 5. Conclusions

In conclusion, our 3D brain slice injury model was successful as a tool for testing a biomaterial implantation therapy, highlighting the use of complex organotypic models for biomaterial testing, to close the gap between simple in vitro 2D models and complex in vivo models. The slices require technically simple procedures and limited training, and are highly cost-effective and humane versus live animal models. Accordingly, they offer the capacity to become a standardised screening model to evaluate neuromaterials, prior to therapeutic testing in live animal models. Detecting the failure of therapeutics in organotypic models could limit therapeutic failure in live animals, highlighting the importance of biomaterial screening in reliable, pathomimetic and neuromimetic in vitro models, and ensuring that only the most promising interventions progress into live animal testing. 

## Data Availability

The original contributions presented in the study are included in the article/Supplementary Materials, further inquiries can be directed to the corresponding author.

## Supplementary Materials

The following supporting information can be downloaded at: https://www.mdpi.com/article/10.3390/jfb15120362/s1, Figure S1: Quantification of GFAP optical density from the astrocytic edge and into the slice; Figure S2: Representation of the microglial shapes translating to a cell roundedness index.
