# Supplementary material for: Neural Cell Interactions with a Surgical Grade Biomaterial Using a Simulated Injury in Brain Organotypic Slices
Source: J Funct Biomater. 2024 Nov 30;15(12):362. doi: 10.3390/jfb15120362 (PMC11678806; doi:10.3390/jfb15120362)
Supplement: Supplementary file 1 [file jfb-15-00362-s001.zip › jfb-3287865-supplementary.pdf]

## Supplementary figures

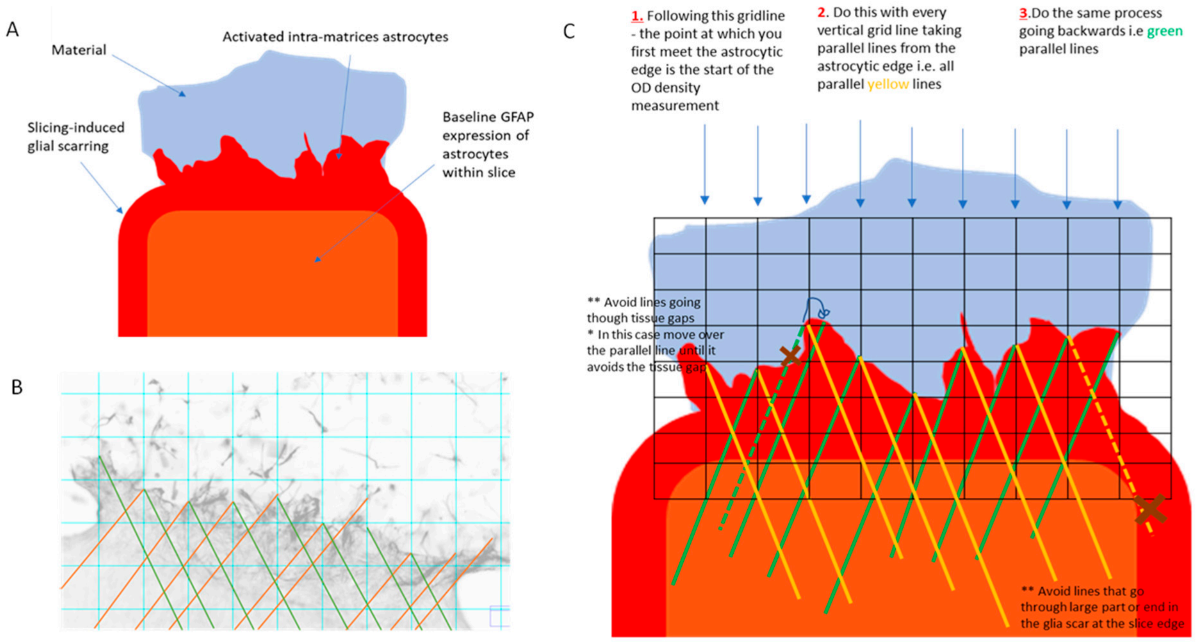


***Supplementary Figure S1. Quantification of GFAP optical density from the astrocytic edge and into the slice.*** *(**A**) A diagram of injury + DG spinal cord slice astrocytic response. (**B**) An example of an GFAP stained injury + DG image converted to an inverted 8-bit image type on ImageJ (lines represent where optical density (OD) measurements were taken). (**C**) Explains the quantification technique process once the grid was overlaid. Values generated by Image-J were extracted into excel, and a fold change for each data set plotted. The same method was used with injury-only tissue slices.*

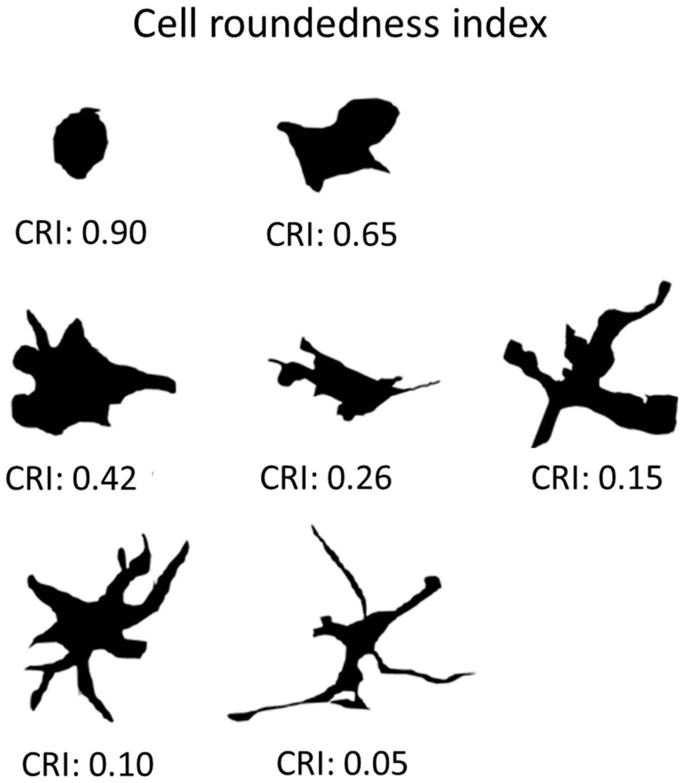


***Supplementary Figure S2. Representation of the microglial shapes translating to a cell roundedness index.***
